# Transient Depletion of CD169^+^ Cells Contributes to Impaired Early Protection and Effector CD8^+^ T Cell Recruitment against Mucosal Respiratory Syncytial Virus Infection

**DOI:** 10.3389/fimmu.2017.00819

**Published:** 2017-07-13

**Authors:** Dong Sun Oh, Ji Eun Oh, Hi Eun Jung, Heung Kyu Lee

**Affiliations:** ^1^Biomedical Science and Engineering Interdisciplinary Program, Korea Advanced Institute of Science and Technology (KAIST), Daejeon, South Korea; ^2^Laboratory of Host Defenses, Graduate School of Medical Science and Engineering, KAIST, Daejeon, South Korea; ^3^KAIST Institute for Health Science and Technology, KAIST, Daejeon, South Korea

**Keywords:** alveolar macrophage, respiratory syncytial virus, type I interferon, CD169, CXCL9/10

## Abstract

Respiratory syncytial virus (RSV) is a major cause of respiratory viral infections in infants and children. Alveolar macrophages (AMs) play a crucial role in combatting airborne pathogens, strongly express CD169, and are localized in the lung alveoli. Therefore, we used CD169-diphtheria toxin receptor (DTR) transgenic mice to explore the roles of CD169^+^ cells in immune responses to mucosal RSV infection. The administration of diphtheria toxin to CD169-DTR mice induced specific AM depletion and reduced the recruitment of Ly6C^hi^ monocytes. Notably, CD169^+^ cell depletion reduced levels of innate cytokines, such as interferon-β, IL-6, and TNF-α, in bronchoalveolar lavage fluid during RSV infection without affecting the production of proinflammatory chemokines. Moreover, the depletion of CD169^+^ cells increased the recruitment of inflammatory cells to the lung during the early stage of RSV infection, although not during the later stages of RSV infection. Furthermore, the depletion of CD169^+^ cells reduced the recruitment of effector CD8^+^ T cells to the lungs after RSV mucosal infection. Our findings suggest that modulating the number of CD169^+^ cells to enhance immune responses to RSV infection may be useful as a new therapeutic strategy.

## Introduction

Respiratory syncytial virus (RSV) is a major respiratory pathogen and the leading cause of bronchiolitis and infant hospital admissions throughout the developed world (1–3). In addition, RSV is a primary cause of opportunistic respiratory infections in elderly and immunocompromised patients (4). Despite the many attempts to develop an RSV vaccine, none are currently licensed, likely due to the lack of sufficient understanding of the host antiviral response to RSV (5, 6). When infecting the lung mucosa, RSV directly or indirectly targets various cell types, including primary epithelial cells, alveolar macrophages (AMs), and dendritic cells (DCs) (7, 8). Thus, the identification of candidate cells to target for vaccine delivery may result in an effective strategy to generate anti-RSV immunity.

Alveolar macrophages localize in the alveoli where they play crucial roles in the maintenance of lung homeostasis and the clearance of airway dust, constituting a front line of defense against airborne pathogens such as RSV (9, 10). Several studies using clodronate liposomes to induce irreversible damage and apoptosis in AMs have shown that AMs play a role in RSV infection (11–13). However, although clodronate liposomes can be used to deplete AMs, they also deplete other lung phagocytic cells, such as DCs, neutrophils, and monocytes (14–16). Therefore, more specific models of AM depletion are needed to study the roles of AMs in RSV mucosal infection.

Type I interferons (IFNs) are important cytokines in the innate immune response to viral infection (17) that modulate the expression of numerous genes involved in host defense and initiate the adaptive immune response to RSV infection (18–21). Recently, type I IFNs and their downstream signaling pathways were shown to be critical for the induction of lung inflammation in response to RSV infection in mice (22). Furthermore, the mitochondrial antiviral-signaling protein (MAVS)-dependent RIG-I-like receptors pathway in AMs was found to be required for type I IFN production in the lungs of RSV-infected mice (23). CD169, also known as Siglec-1 or sialoadhesin, is a sialic acid- and glycoprotein-binding adhesion molecule expressed by AMs (24). The administration of diphtheria toxin (DT) to CD169-diphtheria toxin receptor (DTR) transgenic mice causes the effective and specific ablation of AMs (25), making this a useful model in which to study the roles of these cells.

Respiratory syncytial virus-specific effector CD8^+^ T cells have been reported to play an essential role in recovery from RSV infection (26); however, RSV possesses a mechanism that suppresses lung CD8^+^ T cell effector and memory activity (27). CXCL9/10, which are IFN-inducible chemokines, are required for effector CD8^+^ T cell recruitment into sites of infection and for the amplification of immune responses (28, 29). Thus, to overcome RSV-mediated CD8^+^ T cell suppression, it is important to identify the cell type responsible for CXCL9/10 secretion. Previously, CXCL9/10 was found to be secreted mainly by lung epithelial cells (30, 31); however, little is known regarding the role of infiltrating immune cells in CXCL9/10 production and effector CD8^+^ T cell recruitment to the lungs during mucosal RSV infection.

Here, we elucidate the roles of CD169^+^ cells in antiviral immune responses against mucosal RSV infection. DT administration to CD169-DTR mice was found to induce specific AM depletion and to reduce the recruitment of Ly6C^hi^ monocytes. Notably, the depletion of CD169^+^ cells diminished the secretion of innate cytokines such as IFN-β, IL-6, and TNF-α in bronchoalveolar lavage (BAL) fluid during RSV infection without affecting proinflammatory chemokine production. The depletion of CD169^+^ cells increased the recruitment of inflammatory cells to the lungs during the early stage of RSV infection, but not during later stages of RSV infection. Finally, we found that, although depletion of CD169^+^ cells reduced CXCL9/10 production and recruitment of effector CD8^+^ T cells to the lungs after RSV mucosal infection, CD169^+^ cells were dispensable for CD8^+^ T cell priming against RSV infection.

## Materials and Methods

### Ethics Statement

All animal procedures met the relevant legal and ethical requirements and were in accordance with the guidelines and protocols (KA2013-55) for rodent research approved by the Institutional Animal Care and Use Committee of KAIST.

### Mice

CD169-DTR (Siglec1<tm1 (HBEGF) Mtka>) mice were kindly provided by Dr. Masato Tanaka of the Tokyo University of Pharmacy and Life Sciences (Tokyo, Japan). We used heterozygous CD169-DTR mice and wild-type (WT) littermates as controls in our experiments. The mice were bred and housed in an SPF facility at the KAIST.

### RSV Infection *In Vivo*

As previously reported (32), we depleted CD169^+^ cells in 8- to 15-week-old male WT and CD169-DTR mice by treating the mice intraperitoneally with 40 ng/g DT (Sigma Aldrich, MO, USA) 1 day before infection with RSV. We grew the A2 strain of RSV, kindly provided by Dr. Jun Chang (Ewha Womans University, Seoul, Korea), in HEp-2 cells. We titrated the virus for infectivity as previously described (33). We anesthetized the mice with a mixture of 80 mg/kg ketamine (Youhanyanghaeng, Seoul, Korea) and 16 mg/kg xylazine (BAYER Korea, Ansan, Korea) in PBS (Genedepot, TX, USA) and then inoculated them intranasally with 1.0 × 10^7^ plaque-forming units of RSV.

### Single-Cell Isolation

We analyzed immune cells in lungs of mice according to previously described methods (34). Briefly, lungs were removed from mice, minced into small pieces, and then digested with a mixture of 2 mg/ml collagenase IV (Worthington Biochemical Corp., NJ, USA) and 30 µg/ml DNase I (Roche, Basel, Switzerland) in Dulbecco’s modified Eagle’s medium (Welgene, Daegu, Korea) for 30 min at 37°C. Digested lung tissues were then passed through 70-µm cell strainers prior to Percoll^®^ (GE Healthcare, Uppsala, Sweden) density-gradient (30/70%) centrifugation to isolate the leukocytes. Red blood cells were lysed using an in-house ACK lysis buffer.

### Flow Cytometry

Single-cell suspensions were pretreated with anti-CD16/32 (clone 2.4G2; TONBO Biosciences, CA, USA) antibody to block Fc receptors prior to staining with anti-mouse CD3ε (clone 145-2C11), CD11b (clone M1/70), CD11c (clone N418), CD44 (clone IM7), CD62L (clone MEL-14), CD64 (clone X54-5/7.1.1), Ly6C (clone AL-21), Ly6G (clone 1A8), Siglec-F (clone E50-2440), and Siglec H (clone 551) (BD Biosciences, CA, USA), CD103 (clone 2E7) and MHC Class II (clone M5/114.15.2) (eBioscience, CA, USA), CD4 (clone GK1.5) and CD8α (clone 53-6.7) (TONBO Biosciences, CA, USA), CD45.2 (clone 104) (Biolegend, CA, USA), and CD169 (clone MOMA-1; AbD Serotec, NC, USA). DAPI (Invitrogen, CA, USA) or propidium iodide (eBioscience) was used to exclude dead cells.

To analyze RSV-specific CD8^+^ T cells, we prepared H-2Db tetramers specific for the RSV M_187–195_ peptide (NAITNAKII) using the protocol of the NIH Tetramer Core Facility. We stained the cells with APC-labeled tetramers prior to surface staining. Intracellular cytokine staining was performed based on a previously described method (35). Briefly, we incubated the cells with 50 ng/ml phorbol myristate acetate (Sigma Aldrich), 1 µg/ml ionomycin (Sigma Aldrich), and 2 µM GolgiStop (BD Biosciences) for 5 h at 37°C. For the RSV-specific restimulation of CD8^+^ cells, we cultured the cells in the presence of 5 µg/ml M_187–195_ peptide (NAITNAKII) and 2 µM GolgiStop for 5 h. We then stained the cells with anti-mouse CD4, CD8α, CD11b, CD44, and CD45.2 prior to fixation and permeabilization using the Cytofix/Cytoperm Kit (BD Biosciences) according to the manufacturer’s protocol. We used IFN-γ antibody (clone XMG1.2; BD Biosciences) to detect intracellular cytokines. All samples were analyzed on an LSR Fortessa cell analyzer (BD Biosciences), and data were analyzed using the FlowJo software (Tree Star, OR, USA).

### Cytometric Bead Array and ELISA

To perform BAL on the mice, RSV-infected mice were euthanized at indicated time points after infection. The thorax of each mouse was then opened, and a 20 GA × 1.16 IN (1.1 mm × 30 mm) I.V. catheter (Sewon Medical, Seoul, Korea) was introduced into the trachea at the cricothyroid membrane. The trachea was then flushed with 500 µl PBS, and the resulting fluid was aspirated. Collected BAL fluids were stored at −80°C. IFN-β and TNF-α (Biolegend, CA, USA), IL-6 (BD Biosciences), and CXCL9 and CXCL10 (R&D systems, MN, USA) were then measured in the BAL fluid samples using ELISA kits according to the manufacturer’s protocols. To measure the proinflammatory chemokines in the BAL fluids, the LEGENDplex™ bead-based immunoassay (Biolegend) was utilized according to the manufacturer’s instructions.

### Quantitative RT-PCR

To isolate the total RNA from mock-infected or RSV-infected, DT-treated WT and CD169-DTR mice, the lungs were removed from the mice and homogenized in a bullet blender (Next Advance, New York, NY, USA) and RNA was isolated using an RNeasy Mini kit (Qiagen, CA, USA) according to the manufacturer’s protocol. cDNA was synthesized using NobleZyme™ M-MLV reverse transcriptase (Noble Bio, Suwon, Korea) according to the manufacturer’s instructions. Quantitative PCR was performed using the CFX96 real-time PCR system (Bio-Rad, CA, USA) with SYBR Green-based quantification (Toyobo, Osaka, Japan). The following gene-specific forward (F) and reverse (R) primers were used (36): *Oas1* (F) GCCTGATCCCAGAATCTATGC and (R) GAGCAACTCTAGGGCGTACTG; *Isg15* (F) GGTGTCCGTGACTAACTCCAT and (R) TGGAAAGGGTAAGACCGTCCT; and *Hprt* (F) GTTGGATACAGGCCAGACTTTGTTG and (R) GAGGGTAGGCTGGCCTATTGGCT. PCR results were normalized against *Hprt* and are displayed as the fold difference relative to the mock-infected WT control mice.

### RSV Titers in the Lungs

Previously published procedures (33) were used to quantitate RSV titers in lungs. Briefly, RSV-infected mice were euthanized and lungs were collected and stored in PBS prior to processing through 70-µm cell strainers and collection of the supernatants. The RSV titers in the supernatants were measured using a plaque assay on HEp-2 cell monolayers.

### Statistical Analysis

Data are expressed as the mean ± SE. Differences between groups were analyzed using Student’s *t*-tests. All statistical analyses were performed using the GraphPad Prism 7 software (GraphPad). Differences were considered statistically significant when *P* < 0.05.

## Results

### Depletion of AMs and Reduction of Monocyte Recruitment following DT Administration to CD169-DTR Mice

**Figure 1:**
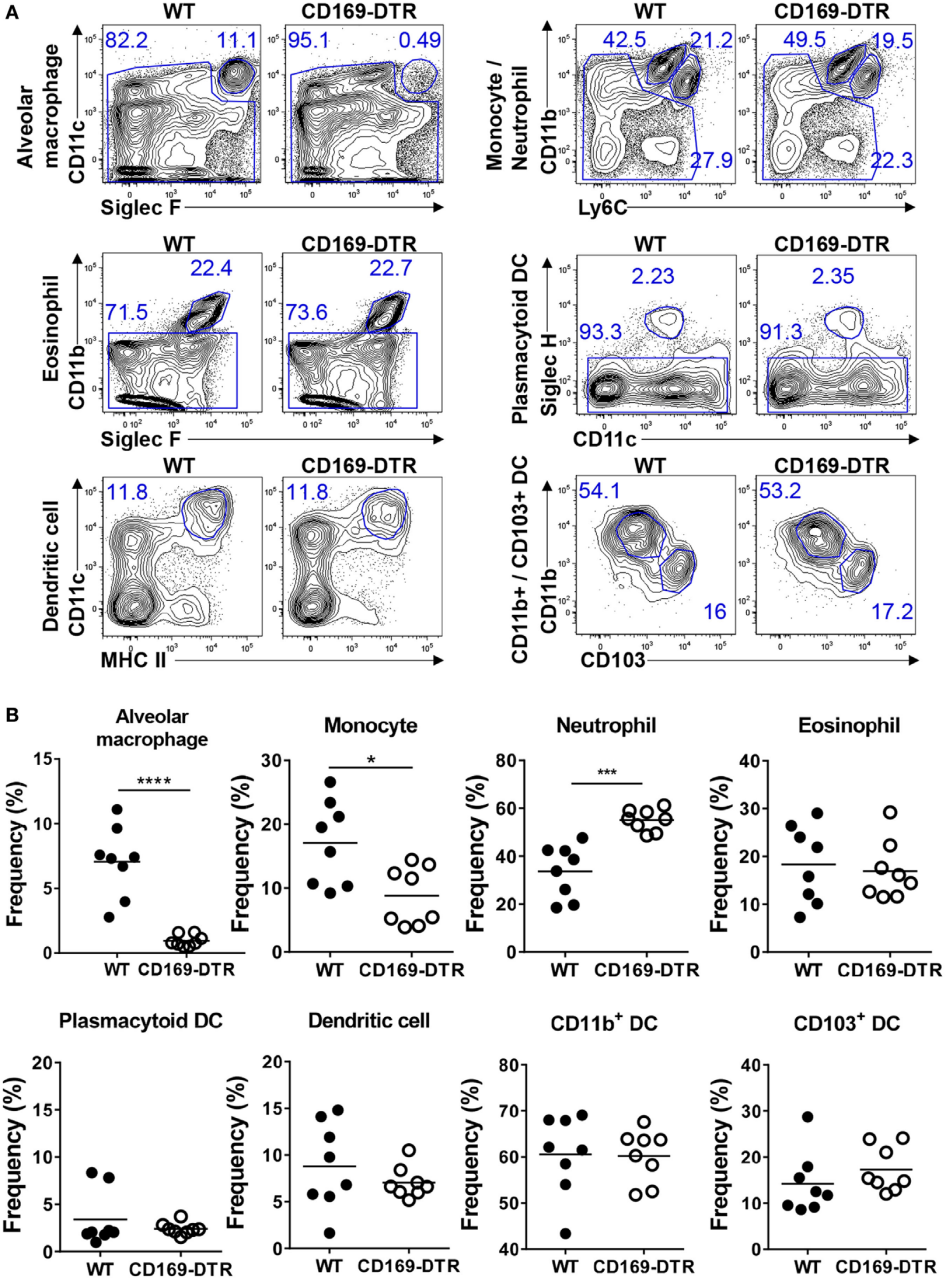
Diphtheria toxin (DT) administration to CD169-diphtheria toxin receptor (DTR) mice induces alveolar macrophage (AM) depletion and reduces monocyte recruitment. **(A)** Lung cells isolated from wild-type (WT) and CD169-DTR mice 1 day after DT treatment were stained with the indicated antibodies and analyzed by flow cytometry. AMs, monocytes, neutrophils, eosinophils, plasmacytoid dendritic cells, dendritic cells (DCs), CD11b^+^ DCs, and CD103^+^ DCs were defined by their surface marker expression. The gating strategy is described in Figure S1 in Supplementary Material. **(B)** Results are shown as dot graphs. Each dot represents an individual mouse (*n* = 8 in each group). Statistically significant differences are indicated: *P < 0.05, ***P < 0.001, ****P < 0.0001. Data are representative of three independent experiments.

CD169 is a known marker of AMs (24), and a previous study showed that DT administration to CD169-DTR mice results in significant AM depletion and induces slight neutrophil recruitment to the lungs but does not contribute to disease in infection experiments (25). We first verified that DT treatment depletes AMs in CD169-DTR mice and determined whether it also affects other myeloid cells in the lungs. Flow cytometric analysis of the lung immune cells (see Figure S1 in Supplementary Material for the gating strategy) from DT-treated WT and CD169-DTR mice revealed significant AM depletion and slightly decreased levels of Ly6C^hi^ monocytes in the CD169-DTR mice. Furthermore, DT treatment increased the neutrophil levels in the lungs of the mice but did not affect the eosinophils, pDCs, CD11b^+^ DCs, or CD103^+^ DCs (Figures 1A,B). Next, we confirmed that T cells, NK cells, monocytes, neutrophils, and other myeloid cells in mouse lungs do not express CD169 (Figure S2 in Supplementary Material). These results suggest that AMs may be involved in the recruitment of Ly6C^hi^ monocytes to the lungs in steady-state conditions. Together, our results suggest that the DT treatment results in an at least 95% depletion of AMs and partially affects the recruitment of monocytes to the lungs of CD169-DTR mice.

### CD169^+^ Cell Depletion Diminishes Innate Cytokine Levels in BAL Fluids during RSV Infection

**Figure 2:**
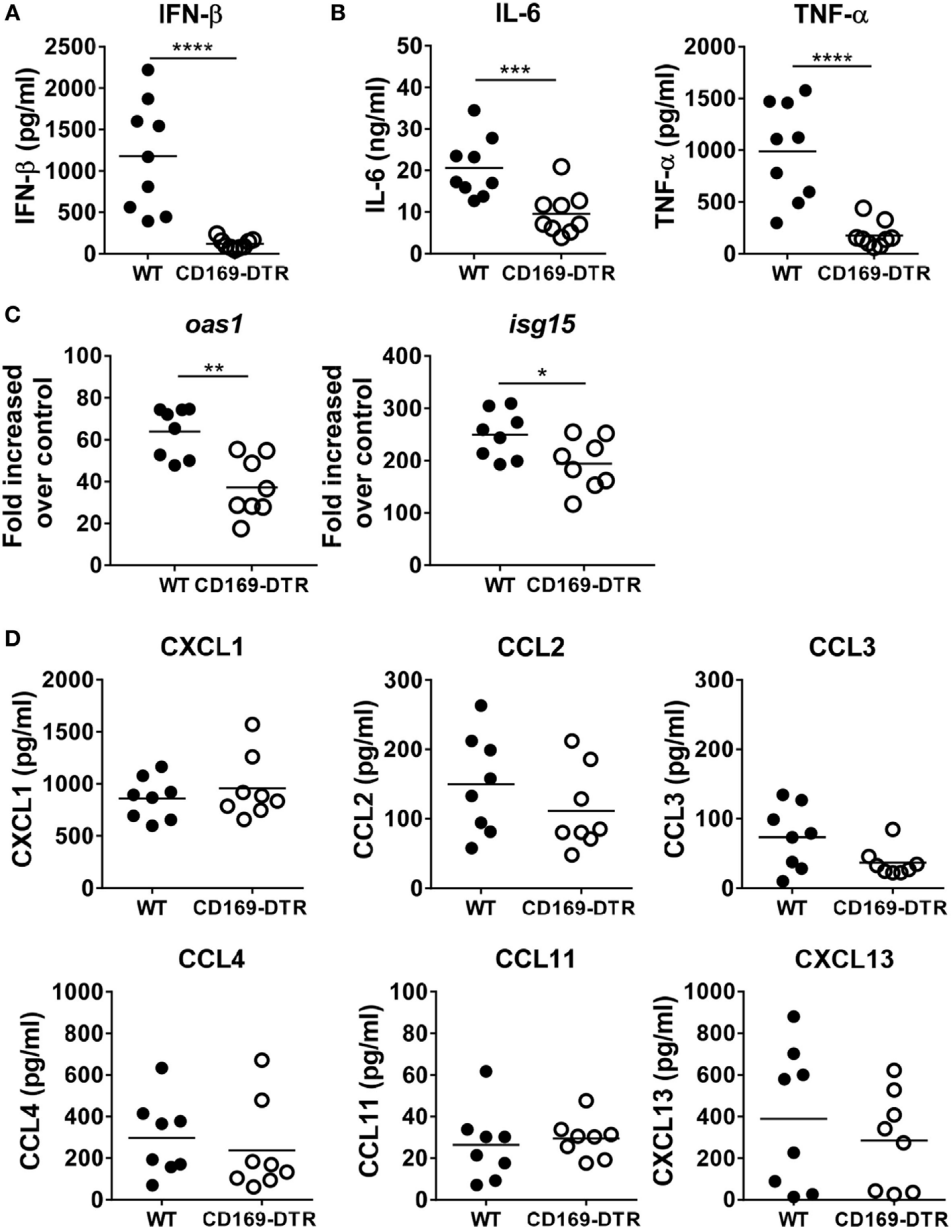
CD169^+^ cell depletion diminishes innate cytokine secretion into bronchoalveolar lavage (BAL) fluid during respiratory syncytial virus (RSV) infection. At the 9 h later of RSV infection, BAL fluid was collected from diphtheria toxin (DT)-treated wild-type (WT) and CD169-diphtheria toxin receptor (DTR) mice. The levels of interferon (IFN)-β **(A)** and IL-6 and TNF-α **(B)** were measured by ELISA. Each dot represents an individual mouse (*n* = 9 in each group). **(C)** At the 1 day later of RSV infection, the levels of *Oas1* and *Isg15* mRNA expression in lung tissues from DT-treated WT and CD169-DTR mice were measured using real-time quantitative PCR. *Hprt* was used as an internal control. **(D)** At the 9 h later of RSV infection, the mouse chemokines CXCL1, CCL2, CCL3, CCL4, CCL11, and CXCL13 were monitored using a cytometric bead array. Each dot represents an individual mouse (*n* = 8 in each group). Statistically significant differences between groups are indicated: *P < 0.05, **P < 0.01, ***P < 0.001, ****P < 0.0001. Data are representative of three independent experiments.

During RSV mucosal infection in mice, IFN-α/β receptor signaling plays a critical role in inducing lung inflammation and limiting viral infection (22), and AMs are major producers of type I IFNs (23). To confirm this, we measured IFN-β, a type I IFN, and the proinflammatory cytokines IL-6 and TNF-α by ELISA in BAL fluids from RSV-infected WT and CD169-DTR mice. Similar to previous results, levels of IFN-β, IL-6, and TNF-α were significantly reduced in BAL fluid from CD169-DTR mice (Figures 2A,B). Furthermore, the mRNA levels of *Oas1* and *Isg15*, two IFN-stimulated genes, were decreased in the RSV-infected lung tissues of the CD169-DTR mice (Figure 2C). Next, we evaluated the levels of proinflammatory chemokines, which are important in inflammatory cell recruitment to the lungs and extravasation into the airways. We found no significant differences in the levels of CXCL1, CCL2, CCL3, CCL4, CCL11, or CXCL13 in the BAL fluid from WT and CD169-DTR mice following RSV infection (Figure 2D). Our results suggest that CD169^+^ cells are important for both early type I IFN secretion and proinflammatory cytokine secretion but are dispensable for proinflammatory chemokine production.

### CD169^+^ Cell Depletion Increases Lung Recruitment of Inflammatory Cells Exclusively during the Early Stage of RSV Infection

**Figure 3:**
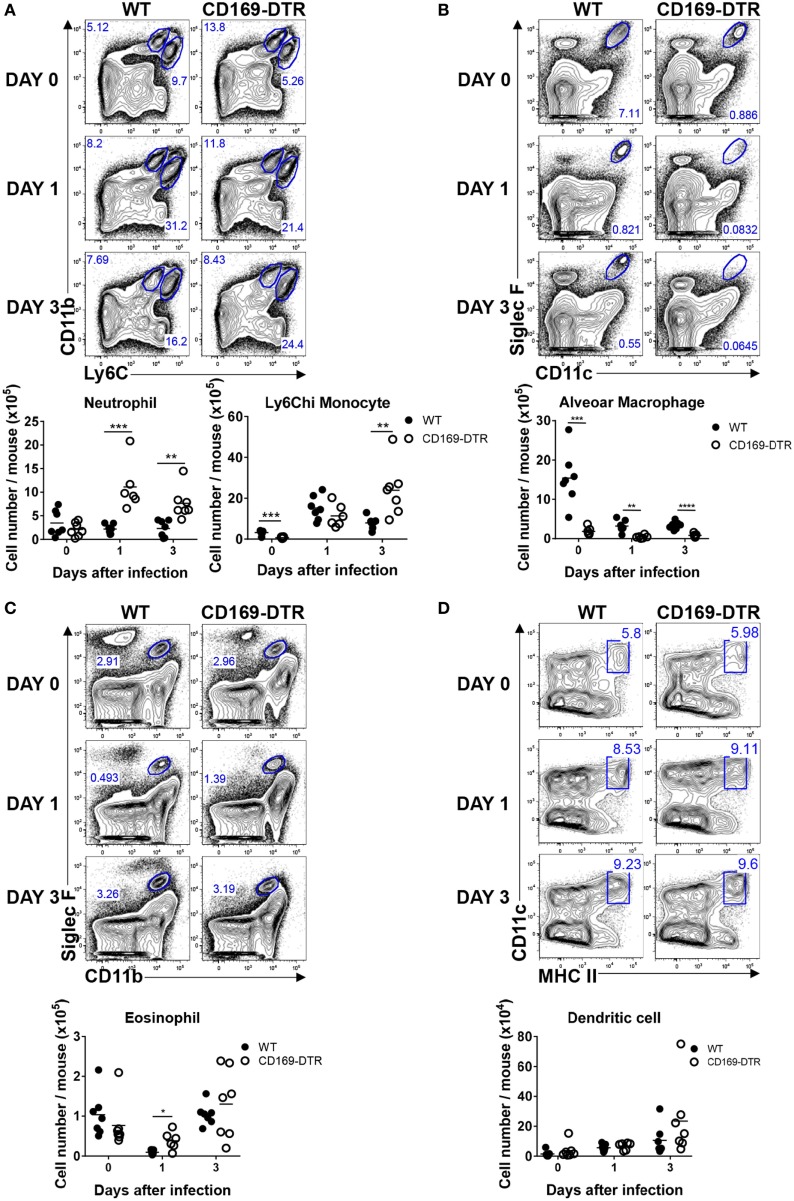
CD169^+^ cell depletion increases the lung recruitment of inflammatory cells during early respiratory syncytial virus (RSV) infection. At the indicated time points during RSV infection, the lungs of diphtheria toxin-treated wild-type (WT) and CD169-diphtheria toxin receptor (DTR) mice were collected, processed, and analyzed by flow cytometry. The numbers of monocytes and neutrophils **(A)**, alveolar macrophages **(B)**, eosinophils **(C)**, and dendritic cells **(D)** indicate the frequencies in gated cells. Cell number results are shown in the graphs; *P < 0.05, **P < 0.01, ***P < 0.001, and ****P < 0.0001. Data are representative of two independent experiments. Each dot represents an individual mouse (*n* = 7 in each group). Statistically significant differences between groups are indicated: *P < 0.05, **P < 0.01, ***P < 0.001, and ****P < 0.0001. Data are representative of two independent experiments.

CD169^+^ AMs undergo apoptosis during RSV infection, an event that may contribute to host defense against intracellular pathogens or viruses (37). In the absence of AMs, virus-infected mice show more severe airway inflammation and enhanced lung pathology (25), suggesting that AMs play an important role in the regulation of the inflammatory response to virus infection. To understand the role of CD169^+^ cells in the trafficking of innate inflammatory cells to the site of infection, we infected DT-treated WT and CD169-DTR mice with RSV, and after the indicated number of days, mice were euthanized and lungs were harvested for analysis by flow cytometry. As the RSV infection progressed, the frequency and number of AMs in the lungs of the mice decreased (Figure 3B). The frequency and number of infiltrating Ly6C^hi^ monocytes and neutrophils (Figure 3A) and eosinophils (Figure 3C) increased in the CD169-DTR mice after RSV infection, whereas the frequency and number of DCs (Figure 3D) were unchanged.

**Figure 4:**
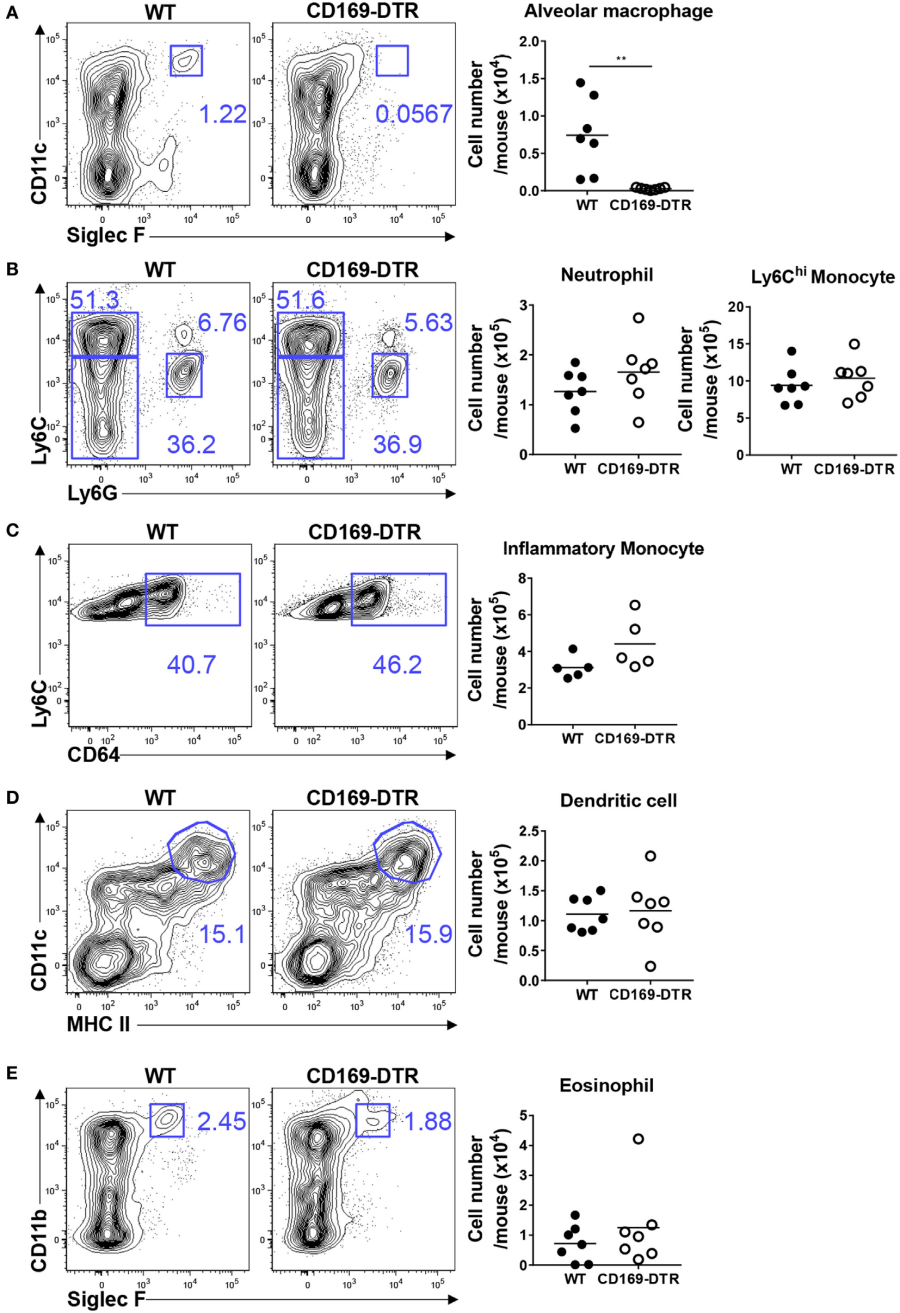
CD169^+^ cell depletion does not affect the lung recruitment of inflammatory cells 5 days after respiratory syncytial virus (RSV) infection. Five days after RSV infection, the lungs of diphtheria toxin-treated wild-type (WT) and CD169-diphtheria toxin receptor (DTR) mice were collected, processed, and analyzed by flow cytometry. The numbers of alveolar macrophages **(A)**, monocytes and neutrophils **(B)**, inflammatory monocytes **(C)**, dendritic cells **(D)**, and eosinophils **(E)** indicate the frequencies in gated cells. Cell number results are shown in the graphs. Each dot represents an individual mouse (*n* = 7 in each group). Statistically significant differences between groups are indicated: **P < 0.01. Data are representative of two independent experiments.

Next, we examined the recruitment of innate inflammatory cells to the site of infection during the later stages of RSV infection. AMs in CD169-DTR mice were still depleted by DT treatment after 5 days of RSV infection (Figure 4A). No substantial differences in the frequency or number of Ly6C^hi^ monocytes and neutrophils (Figure 4B), CD64^hi^ inflammatory monocytes (Figure 4C), DCs (Figure 4D), or eosinophils (Figure 4E) were found between the WT and CD169-DTR mice. Collectively, our results demonstrate that the removal of CD169^+^ cells increases the recruitment of inflammatory cells specifically during the early stage of RSV infection.

### CD169^+^ Cells Are Dispensable for the Adaptive Immune Response against RSV

**Figure 5:**
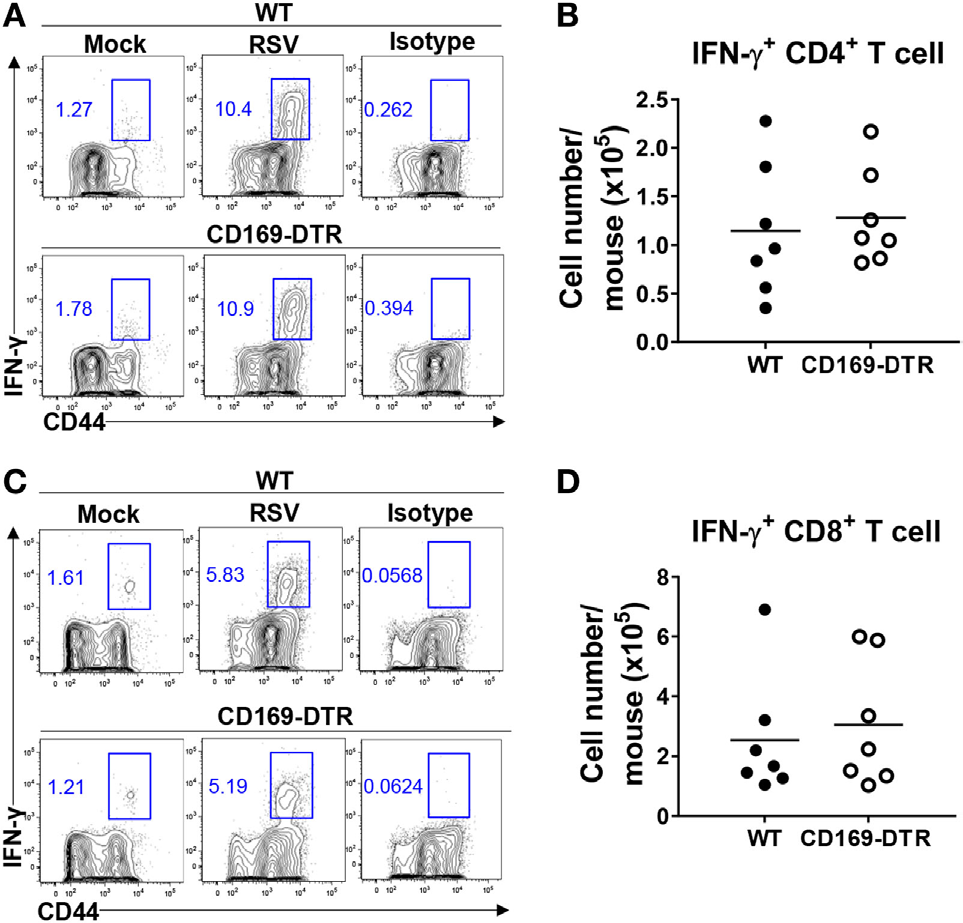
CD169^+^ cell depletion is dispensable for the adaptive immune response against respiratory syncytial virus (RSV). Seven days after RSV infection, interferon (IFN)-γ production by activated CD4^+^ or CD8^+^ T cells from lungs was measured by intracellular cytokine staining after stimulation with PMA and ionomycin (for CD4^+^ T cells) **(A)** or H-2Db-restricted M_187–195_ peptides (for CD8^+^ T cells) **(C)**. The frequencies and absolute cell numbers of CD44^+^ IFN-γ^+^ CD4^+^ **(A,B)** and CD8^+^ **(C,D)** T cells were assessed. Cell number results from RSV-infected mice are shown in the graphs. Each dot represents an individual mouse (*n* = 7 in each group). Data are representative of two independent experiments.

Next, we examined the role of CD169^+^ cells in adaptive immunity to RSV. While adaptive immune responses to RSV infection are required for efficient viral clearance, they also contribute to RSV-induced disease (38). We infected DT-treated WT and CD169-DTR mice with RSV and used intracellular cytokine staining to monitor IFN-γ production from activated lung CD44^hi^ CD4^+^ and CD44^hi^ CD8^+^ T cells after stimulation with PMA/ionomycin (for CD4^+^ T cells) or H-2Db-restricted M_187–195_ peptides (for CD8^+^ T cells). There was no substantial difference in the Th1 response (Figures 5A,B) or the CTL response (Figures 5C,D) between the WT and CD169-DTR mice. Therefore, we concluded that CD169^+^ cells are dispensable for the adaptive immune response against RSV.

### Depletion of CD169^+^ Cells Reduces the Recruitment of Effector CD8^+^ T Cells to the Lungs after RSV Infection

**Figure 6:**
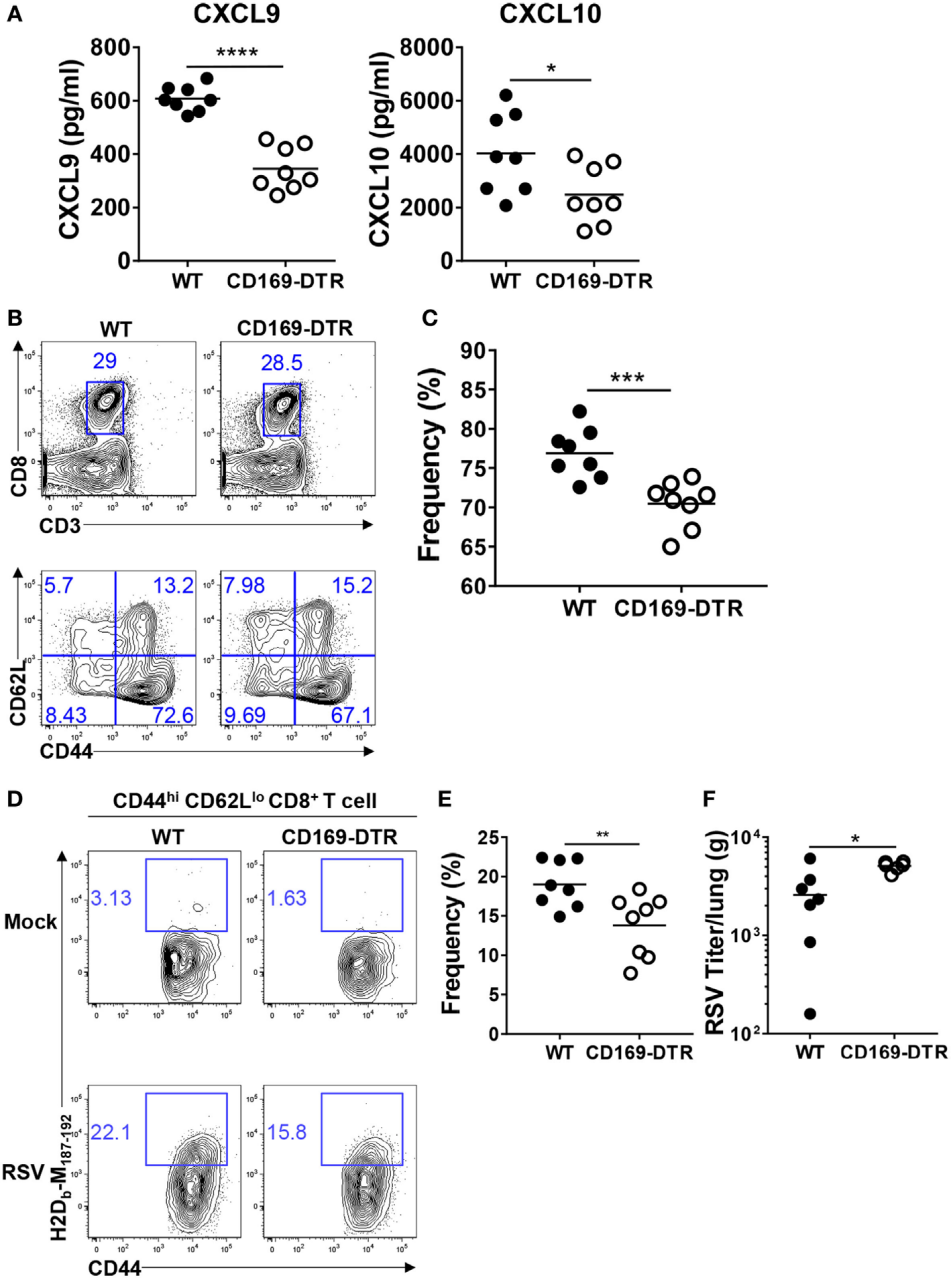
CD169^+^ cell depletion reduces the recruitment of effector CD8^+^ T cells in lungs after respiratory syncytial virus (RSV) infection. At the indicated time points after RSV infection, bronchoalveolar lavage fluid was collected from diphtheria toxin-treated wild-type (WT) and CD169-diphtheria toxin receptor (DTR) mice. The levels of CXCL9 and CXCL10 in the lavage fluids were measured by ELISA **(A)**. Seven days after RSV infection, the frequencies of CD8^+^ T cells **(B)** and CD44^hi^ CD62L^lo^ CD8^+^ T cells **(C)** were assessed by flow cytometry. **(D)** RSV M peptide-specific CD44^hi^ CD62L^lo^ CD8^+^ T cells were detected by flow cytometry. **(E)** Frequencies of CD44^hi^ CD62L^lo^ tetramer^+^ CD8^+^ T cells were quantified from flow cytometry data of RSV-infected mice. **(F)** Five days after infection, RSV viral titers from lung homogenates were measured on HEp-2 cells. Each dot represents an individual mouse (*n* = 8 in each group). Statistically significant differences between groups are indicated: *P < 0.05, **P < 0.01, ***P < 0.001, and ****P < 0.0001. Data are representative of two independent experiments.

An efficient antiviral response induces the recruitment of RSV-specific CD4^+^ and CD8^+^ T cells to the infection site by the CXCL9 and CXCL10 chemokines, which are induced by IFNs and the chemokine receptor CXCR3. CXCL9 and CXCL10 protect the host by reducing the viral load and pathogenesis (30, 39). To understand the role of CD169^+^ cells in the recruitment of effector T cells to the infection site, we measured the CXCL9 and CXCL10 levels in BAL fluid from DT-treated WT and CD169-DTR mice after RSV infection. As shown in Figure 6A, both CXCL9 and CXCL10 were decreased in BAL fluid from CD169-DTR mice following RSV infection.

Next, we confirmed that the reduction of CXCL9 and CXCL10 reduced the entry of effector T cells into the infection site. DT-treated WT and CD169-DTR mice were infected with RSV, and CD44^hi^ CD62L^lo^ CD4^+^ and CD44^hi^ CD62L^lo^ CD8^+^ T cells were assessed by flow cytometry on day 7 after infection. There was no significant difference in the recruitment of CD44^hi^ CD62L^lo^ CD4^+^ T cells to the lungs (Figure S3 in Supplementary Material); however, the recruitment of CD44^hi^ CD62L^lo^ CD8^+^ T cells was decreased in the CD169-DTR mice (Figures 6B,C). Staining with RSV-specific tetramers showed that the frequency of RSV-specific CD44^hi^ CD62L^lo^ CD8^+^ T cells was decreased in the lungs of the RSV-infected CD169-DTR mice (Figures 6D,E).

Finally, we determined whether the effects of the CD169^+^ cell depletion on innate and adaptive immunity influence RSV replication. Our results show that viral clearance was impaired 5 days after RSV infection in CD169-DTR mice (Figure 6F). Taken together, these results show that CD169^+^ cells contribute to the recruitment of effector CD8^+^ T cells to the site of RSV infection and also to the clearance of the virus from the lung mucosa.

## Discussion

In this study, we investigated the roles of CD169^+^ cells in immune responses against mucosal RSV infection. DT administration to CD169-DTR mice was found to specifically induce the depletion of AMs and reduce the recruitment of Ly6C^hi^ monocytes. Notably, CD169^+^ cell depletion diminished the levels of innate cytokines, such as IFN-β, IL-6, and TNF-α, in BAL fluid from RSV-infected mice without affecting the production of proinflammatory chemokines. Moreover, the depletion of CD169^+^ cells increased the recruitment of inflammatory cells to the lungs exclusively during the early stage of RSV infection. Furthermore, the depletion of CD169^+^ cells reduced the recruitment of effector CD8^+^ T cells to the lungs after RSV mucosal infection. However, the CD169^+^ cells were dispensable for the adaptive immune response against RSV.

The lung macrophages comprise AMs in the luminal area of alveoli and interstitial macrophages (IMs) within the lung parenchymal interstitium. IMs do not express Siglec-F or CD169 (40) but show characteristics of pro-fibrotic cells in a bleomycin-mediated lung fibrosis model (41). Moreover, in contrast to AMs, IMs play a role in the production of IL-10 (42). The results from this study demonstrate that lung IMs have a unique role in the lung and alter DC functions to prevent ovalbumin/lipopolysaccharide-induced airway allergies in mice. Further studies are required to elucidate the role of IMs in RSV mucosal infection. However, we established the role of AMs in RSV infection by specifically depleting them by DT administration in a CD169-DTR mouse model. Whereas very long-term administration of DT to these mice promotes reduced erythroblast numbers (43), it does not induce any systemic toxic effect (32, 44). Importantly, our studies were performed after short-term administration with a lower dose of DT compared with previous studies.

Our results suggest that AMs contribute to the recruitment of Ly6C^hi^ monocytes to the lungs in steady-state conditions. Although macrophages can be a source of monocyte chemoattractants (45), DT treatment did not alter the levels of inflammatory chemoattractants in BAL fluid from CD169-DTR mice. Recent research using a murine colitis model showed that CD169^+^ macrophages contribute to the accumulation of colitis-associated CD169^−^ Ly6C^hi^ CD64^lo^ monocytes in the colon mucosa in a CCL8-dependent manner (46). In agreement with this, we found that the levels of CCL8 mRNA in the lungs were decreased in CD169-DTR mice after RSV infection (data not shown). Further research is needed to determine whether AMs are involved in CCL8-dependent monocyte recruitment to the lungs.

Various types of lung cells, such as epithelial cells, macrophages, fibroblasts, DCs, and pDCs, are known to secrete type I IFNs during RSV infection. Alveolar-located DCs send their dendrites trans-epithelially into airspace and assess particulate antigens (47) or RSV virions (48) and have functions to produce various inflammatory mediators, including type I IFNs (49). In addition, alveolar DCs can be infected with RSV *in vitro* or *in vivo* and produce type I IFNs and proinflammatory cytokines (48, 50). Similarly, AMs are known to produce type I IFNs after virus infection (51–53) and are known to be resistant to RSV replication, which may help to sustain their activity (54). Our results support previous claims that AMs are major IFN producers in the lungs after RSV infection, while some studies suggested that pDCs are the source of type I IFNs during RSV infection (55, 56). Moreover, our results show that AMs contribute to the production of proinflammatory cytokines such as IL-6 and TNF-α during RSV infection. Although primary-airway epithelial cells and AMs have been associated with the production of proinflammatory chemokines (57, 58), our results show that AMs are dispensable for proinflammatory chemokine production during RSV infection, suggesting that airway epithelial cells or other cells are sufficient for their production.

Alveolar macrophages undergo apoptosis in the early phase of RSV infection, resulting in a reduction of the disease severity and the eventual resolution of inflammation (37). Consistent with previous results, RSV infection in our mouse model reduced AM levels, an effect that was sustained even 5 days after infection. Not only do AMs play a crucial role in the maintenance of lung homeostasis and the clearance of airway dust, but also there is increasing evidence indicating that severe pulmonary disease caused by RSV infection in infancy is associated with recurrent wheezing and the development of asthma later in childhood (59, 60). Recent studies indicated that AMs regulate inflammatory immune responses in the airways and that these cells have a critical role in asthma (61). In a mouse model of house dust mite-induced asthma in which AMs are depleted using clodronate liposomes, Th2 cytokines, such as IL-4, IL-5, and IL-13, and inflammatory cytokines and eosinophil recruitment were increased in BAL fluid, suggesting an immunosuppressive role of AMs (62). Our results suggest a possible mechanism of asthma development following RSV infection in which the failure of homeostasis is due to the induction of AM apoptosis.

During the early phase of RSV infection, neutrophil and monocyte recruitment, which is characteristic of virus-mediated inflammation, was slightly increased in the lungs of CD169-DTR mice. However, the recruitment of these cells was comparable between the WT and CD169-DTR mice during the later stages of RSV infection. This suggests that AMs are required for protection against RSV-induced tissue injury or excessive inflammatory signaling in the early phase of the infection, but their importance decreases at later time points due to RSV-induced apoptosis. The relationship between AMs and RSV-induced eosinophilia, which have been proposed to play roles in the pathogenesis of lower respiratory tract disease (63, 64), is unclear. Our data suggest that AMs are required to resolve eosinophilia during the early stage of RSV infection but not during the later stages. Further studies are needed to determine whether AMs are associated with chemokine production important for eosinophil recruitment.

A previous study noted that MAVS is important for sensing RSV (23). In MAVS-deficient mice, AMs did not produce type I IFNs and there was a defect in the recruitment of inflammatory monocytes to the lungs downstream of type I IFN signaling (23). Our results show that AM depletion reduces the secretion of type I IFN but does not affect the recruitment of CD64^hi^ inflammatory monocytes. This suggests that other factors are involved in the recruitment of CD64^hi^ inflammatory monocytes to the lungs.

While the adaptive T cell immune response to RSV is important for the promotion of viral clearance, a dysregulated T cell response to RSV can induce immunopathology. Macrophage depletion by clodronate liposomes did not affect the recruitment of activated CD4^+^ or CD8^+^ T cells, weight loss, or virus-induced changes in mouse lung function (12). Although AMs suppress the migration (65) and antigen-presentation capacity (66) of DCs and increase the trafficking of antigen to draining lymph nodes (65), our results show that AM depletion in CD169-DTR mice does not affect DCs or their subset frequency and has no effect on the adaptive CD4^+^ and CD8^+^ T cell immune responses to RSV infection. CXCL9 and CXCL10 play important roles in RSV-infected mice by recruiting virus-specific T cells to the lungs and promoting viral clearance (39, 67). Multiple cell populations, including epithelial cells and fibroblasts, may induce the production of CXCL9 and CXCL10 in the airways (68). Moreover, type I IFN production provides additional increases in CXCL9 and CXCL10 production, thereby enhancing the antiviral response (69). Our results newly demonstrate roles of AMs in CXCL9 and CXCL10 production, and as a result, in the recruitment of antigen-specific effector CD8^+^ T cells to the lungs. Furthermore, our results demonstrate that AMs are required for RSV clearance.

Recently, a “prime-and-pull” vaccine strategy was developed to induce local resident memory T cells at an infection site using a model of genital mucosal HSV-2 infection (70). This strategy comprises a conventional vaccination to provoke a systemic T cell responses (i.e., the prime) and administration CXCL9/10 chemokines to the genital tract to recruit activated T cells (i.e., the pull), thereby establishing long-term protective immunity. This strategy provoked more efficient protective T cell responses in the lungs after flu infection (71). The results from our study suggest that AMs are the major producer of CXCL9/10 in mucosal RSV infection. Thus, AMs can be considered novel targets for the development of vaccines designed to provoke more efficient local memory T cell immunity *via* secretion of CXCL9/10.

In conclusion, our study reveals that AMs are responsible for the production of type I IFNs and proinflammatory cytokines and for the recruitment of effector CD8^+^ T cells *via* CXCL9 and CXCL10 production. Furthermore, AMs are required for viral clearance in RSV mucosal infection. Therefore, AMs might provide a novel target for the development of an RSV vaccine using the prime-and-pull strategy to stimulate AMs to induce type I IFN and CXCL9 and CXCL10 secretions (70).

## Ethics Statement

All animal procedures met the relevant legal and ethical requirements and were in accordance with the guidelines and protocols (KA2013-55) for rodent research approved by the Institutional Animal Care and Use Committee of KAIST.

## Author Contributions

DO and HL designed the study. DO, JO, and HJ performed the research. DO and HL conceived the study, analyzed the data, and wrote the manuscript.

## Conflict of Interest Statement

The authors declare that the research was conducted in the absence of any commercial or financial relationships that could be construed as a potential conflict of interest.
