# Supplementary material for: Transient Depletion of CD169+ Cells Contributes to Impaired Early Protection and Effector CD8+ T Cell Recruitment against Mucosal Respiratory Syncytial Virus Infection
Source: Front Immunol. 2017 Jul 13;8:819. doi: 10.3389/fimmu.2017.00819 (PMC5507946; doi:10.3389/fimmu.2017.00819)
Supplement: Supplementary file 1 [file Data_Sheet_1.DOCX]

Supplementary Material

Transient depletion of CD169^+^ cells contributes to impaired early protection and effector CD8^+^ T cell recruitment against mucosal respiratory syncytial virus infection

Dong Sun Oh, Ji Eun Oh, Hi Eun Jung, Heung Kyu Lee^*^

*** Correspondence to:** Corresponding Author: [heungkyu.lee@kaist.ac.kr](mailto:heungkyu.lee@kaist.ac.kr)

**Supplementary Figure 1.** **Gating strategy for identifying lung cell populations**

Lung cells were obtained by collagenase IV and Dnase I digestion and stained with the indicated cell-surface molecules. The represented gates were used to identify Ly6C^hi^ monocytes, neutrophils, eosinophils, AMs, conventional DCs, CD103^+^ DC, CD11b^+^ DC, and pDCs. Dead cells were excluded by PI staining. Cell debris were removed by singlet gating and FSC SSC-based gating. To define Ly6C^hi^ monocytes, CD3ε^-^NK1.1^-^B220^-^CD11b^+^Ly6C^hi+^ cells or CD3ε^-^NK1.1^-^B220^-^CD11b^+^Ly6C^hi+^Ly6G^-^ cells were regarded as monocytes. To define neutrophils, CD3ε-NK1.1-B220-CD11b^hi^Ly6C^+^ cells or CD3ε^-^NK1.1^-^B220^-^CD11b^+^Ly6C^+^Ly6G^+^ cells were regarded as neutrophils.

**Supplementary Figure 2.** **CD169 expression in lung cell populations**

Lung cells collected from WT mice were stained with CD169 and each cell-surface marker. CD169 expression (filled dark gray) was measured by flow cytometry. Isotype control (IgG2a) (filled light gray) was used as a control.

**Supplementary Figure 3.** **Unimpaired effector CD4+ T cell recruitment in CD169-DTR mice**

(A) Seven days after RSV infection, CD44^hi^ CD62L^low^ CD4^+^ T cells in the lungs from DT-treated WT and CD169-DTR mice were analyzed by flow cytometry. (B) The frequency of CD44^hi^ CD62L^low^ CD4^+^ T cells from RSV infected mice is shown as a dot graph. Each dot represents an individual mouse ( n = 8 in each group).
