# Supplementary figures and images for: Transient Depletion of CD169+ Cells Contributes to Impaired Early Protection and Effector CD8+ T Cell Recruitment against Mucosal Respiratory Syncytial Virus Infection
Source: Front Immunol. 2017 Jul 13;8:819. doi: 10.3389/fimmu.2017.00819 (PMC5507946; doi:10.3389/fimmu.2017.00819)

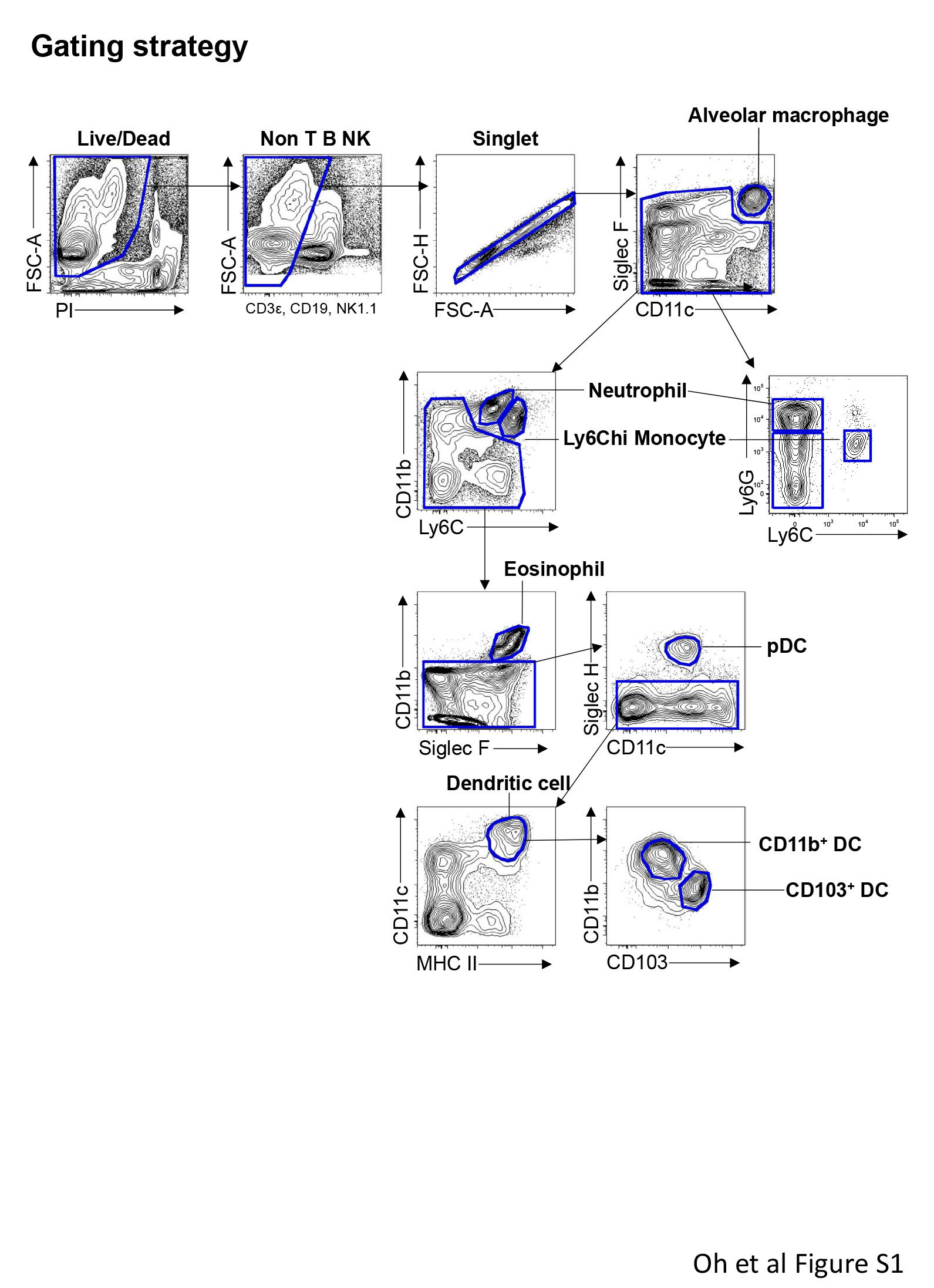

Supplement: Supplementary file 2 [file Image_1.TIF]

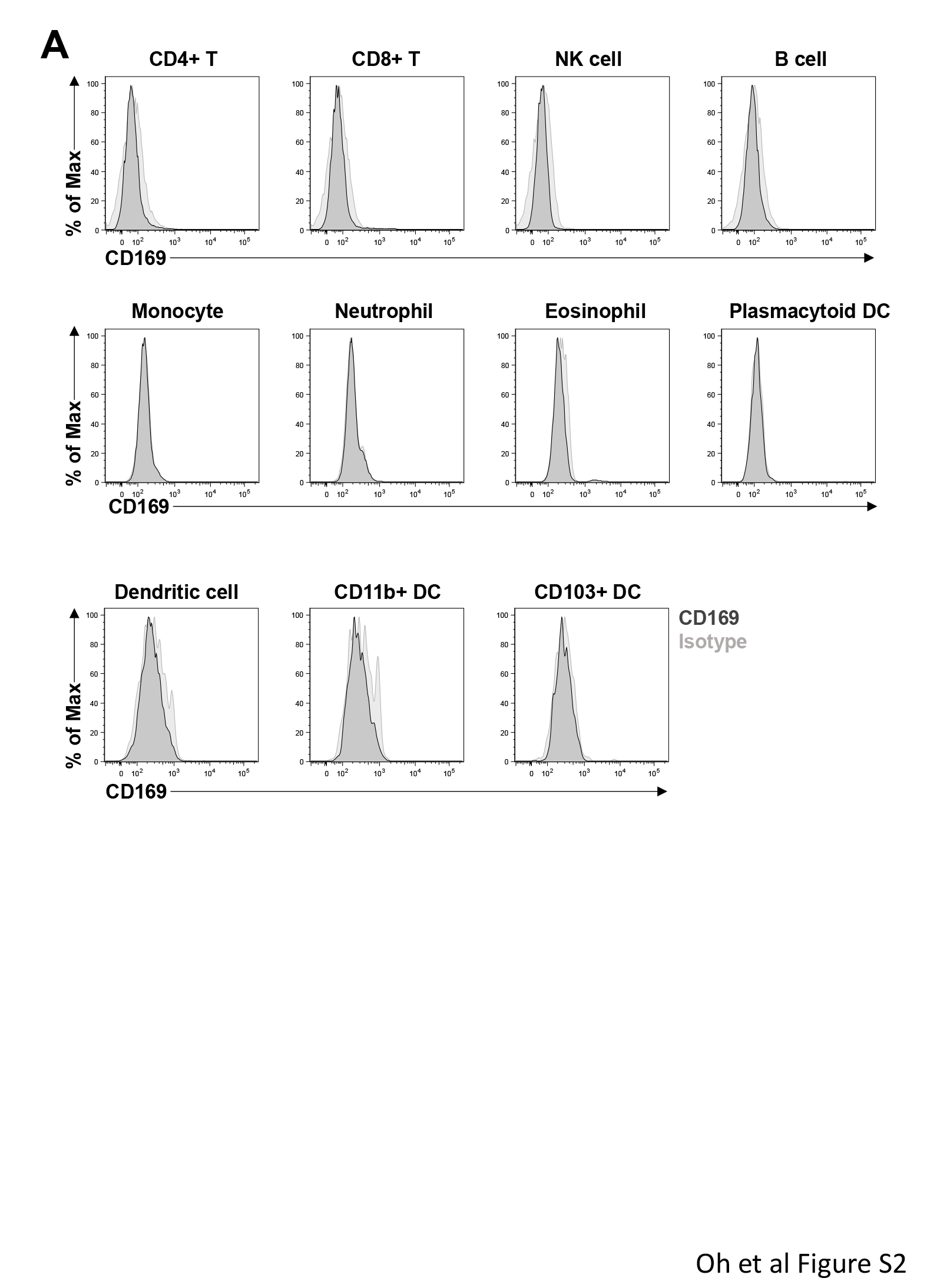

Supplement: Supplementary file 3 [file Image_2.TIF]

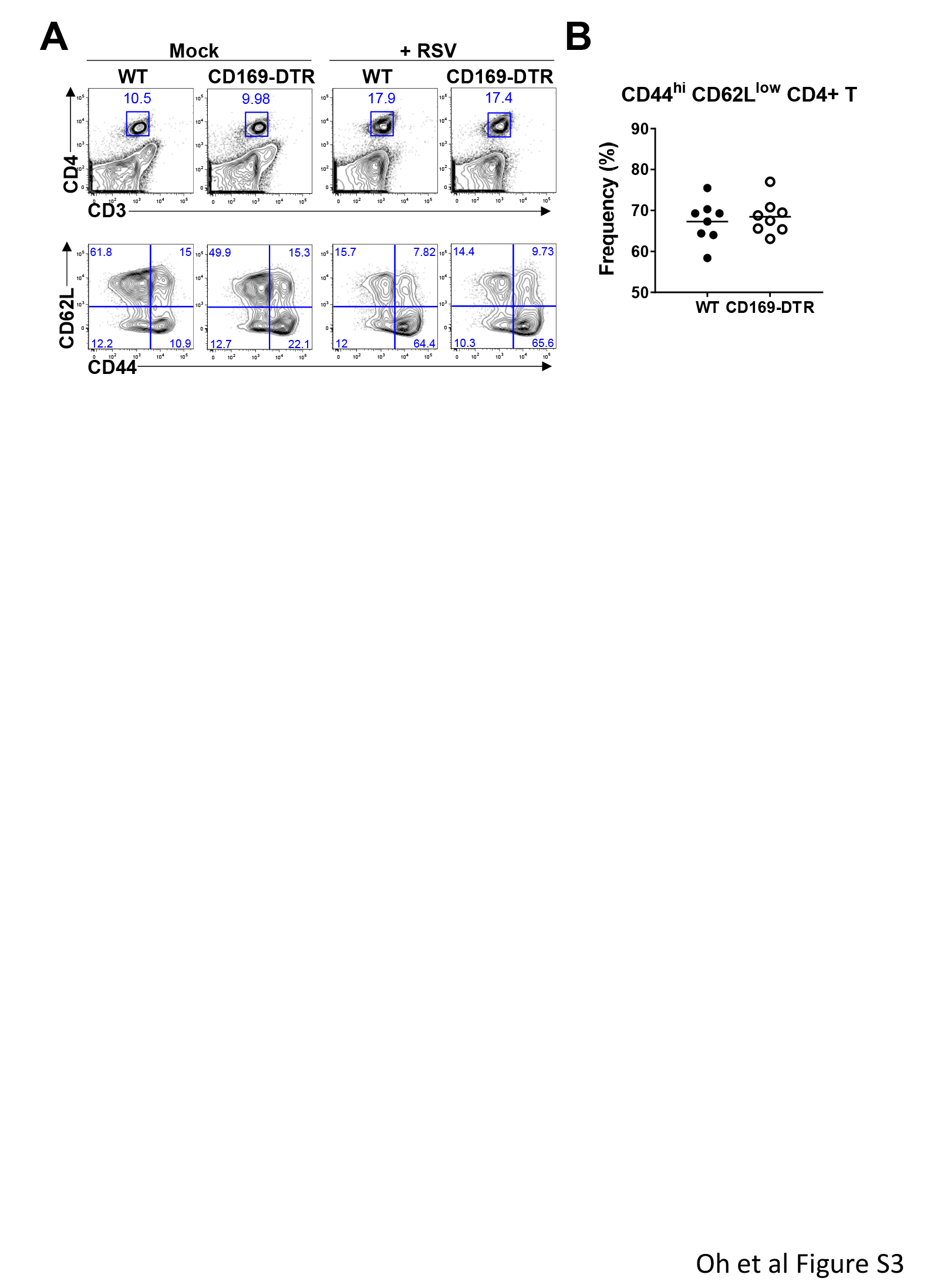

Supplement: Supplementary file 4 [file Image_3.TIF]
